# Iterative Cytoreductive Surgery and HIPEC for Peritoneal Metastases from Primary Appendiceal and Colorectal Cancers: An Observational Study †

**DOI:** 10.3390/cancers17122014

**Published:** 2025-06-17

**Authors:** Andrew M. Fleming, Owen M. Clark, Jaewon J. Lee, Kristen Dougherty, Leah E. Hendrick, Jordan Raine, Ian Solsky, Paxton V. Dickson, Evan S. Glazer, David Shibata, Elizabeth Gleeson, Gitonga Munene, Jeremiah L. Deneve

**Affiliations:** 1 Department of Surgery, The University of Tennessee Health Science Center, Memphis, TN 38163, USAeglazer@uthsc.edu (E.S.G.);; 2 Department of Surgery, The University of North Carolina, Chapel Hill, NC 27599, USAjdeneve@unc.edu (J.L.D.); 3 Division of Gastrointestinal Oncology, H. Lee Moffitt Cancer Center, Tampa, FL 33612, USA; 4 Department of Surgery, The University of Tennessee Health Science Center, Chattanooga, TN 37403, USA; jraine@uthsc.edu; 5 Department of Surgery, Western Michigan University, Kalamazoo, MI 49008, USA

**Keywords:** HIPEC, CRS/HIPEC, cytoreduction, repeat, recurrence, peritoneal carcinomatosis

## Abstract

**Background**: Peritoneal relapse after cytoreduction and hyperthermic intraperitoneal chemotherapy (CRS/HIPEC) is common. Repeat CRS/HIPEC offers the potential for long-term survival in the appropriately selected patient. **Methods**: We performed a retrospective review of a single institution database to assess perioperative outcomes after repeat CRS/HIPEC for appendiceal (pAC) and colorectal (pCRC) cancers. Kaplan–Meier and Cox estimates were used to assess survival. **Results**: Of 157 patients, 103 patients underwent initial CRS/HIPEC for pAC (*n* = 67) or pCRC (*n* = 36) histologies. Twenty-seven pAC patients (27/67, 40%) and 23/36 pCRC patients (63%) developed disease recurrence. Relapsed patients had a higher burden of disease (PCI), operative length and blood loss and received adjuvant chemotherapy (all *p* < 0.05). Nine of the 27 relapsed pAC patients and 5 of the 13 relapsed pCRC patients underwent repeat CRS/HIPEC. The median time to repeat CRS/HIPEC was 18 months (4–26 months), and a CCR-0 and CCR-1 were achieved in 79% and 21%, respectively. The 1-, 3- and 5-year OS for pAC patients who underwent repeat CRS/HIPEC was 88.9%, 88.9% and 77.8%, and the 1- and 3-year OS for pCRC patients was 100% and 25%, respectively. Repeat CRS/HIPEC for pAC was associated with significant improvement in OS (*p* = 0.03), while for pCRC, no significant difference was observed (*p* = 0.99). **Conclusions**: Repeat CRS/HIPEC for isolated peritoneal recurrence is safe and offers the potential for long-term survival. Patient selection is key to ensure optimal cytoreduction when considering repeat CRS/HIPEC.

## Simple Summary

Peritoneal relapse after cytoreduction and hyperthermic intraperitoneal chemotherapy (CRS/HIPEC) is common. Repeat CRS/HIPEC offers the potential for long-term survival in the appropriately selected patient. We examined the outcome for appendiceal and colorectal cancer patients who underwent repeat CRS/HIPEC after isolated peritoneal recurrence. Repeat CRS/HIPEC was safe and associated with the potential for a long-term survival outcome, particularly for appendiceal cancer.

## 1. Introduction

Peritoneal carcinomatosis from primary tumors of the stomach, ovary, appendix, colon and other organs is challenging and typically associated with a poor prognosis. Cytoreduction and hyperthermic intraperitoneal chemotherapy (CRS/HIPEC) has emerged as a treatment approach for peritoneal metastases to improve survival [1]. Most importantly, the ability to perform a complete cytoreduction has the greatest impact on survival [1,2]. Unfortunately, even after optimal cytoreduction, the likelihood of disease recurrence within the peritoneum is high [3,4,5,6].

Relapse within the peritoneum is complicated, and how to most appropriately manage isolated peritoneal recurrence has not yet been determined. Without additional treatment, the natural history for peritoneal carcinomatosis recurrence is progressive weight loss, malnutrition and malignant obstruction [2,7]. Iterative cytoreductive surgery for peritoneal relapse has the potential, in the appropriately selected patient, to offer a meaningful outcome, and several studies have reported single-center experiences of repeat CRS/HIPEC [8,9,10,11,12,13]. The multi-institutional US HIPEC Collaborative series reported the outcomes of over 150 repeat CRS/HIPEC procedures and identified no association with prohibitive complications [14]. Tumor biology, site of recurrence, timing of repeat CRS/HIPEC and safety are all important determinants when considering repeat CRS/HIPEC [15,16,17].

To further study this specific patient population, a retrospective review of a single-center, prospectively maintained CRS/HIPEC database was performed to explore the safety and morbidity for repeat CRS/HIPEC. We specifically investigated the outcomes of appendiceal and colorectal pathologies, as these populations represented the majority of patients treated in our series. Our secondary objective was to compare the overall survival of the repeat CRS/HIPEC cohort with those who did not undergo repeat CRS/HIPEC.

## 2. Methods and Materials

### 2.1. Data Source and Patient Selection

The current analysis represents a retrospective cohort study examining patients undergoing cytoreductive surgery with hyperthermic intraperitoneal chemotherapy (CRS/HIPEC) for appendiceal (pAC) and colorectal (pCRC) peritoneal metastases. This analysis was approved by an institutional review board and was performed in accordance with federal law and the University of Tennessee Health Science Center (UTHSC IRB 17-05211-XP). The current study was performed according to the Strengthening the Reporting of Observational Studies in Epidemiology (STROBE) recommendations [18].

### 2.2. Setting, Patients and Data Sources

All CRS/HIPEC were performed by two surgeons (GM and JD) at a tertiary referral center. Patients with a diagnosis of pAC or pCRC were identified within the electronic medical record from 2011 to 2022. A diagnosis of pAC or pCRC was confirmed by individual review of pathological reports. Patients were excluded if they were treated for other histologies (desmoplastic small round cell tumor, gastric adenocarcinoma, hepatocellular carcinoma, ovarian carcinoma, primary peritoneal mesothelioma, fallopian carcinoma and small bowel adenocarcinoma) or had not completed treatment at the time of analysis. Patients were followed closely pre- and post-operatively in a multidisciplinary oncology clinic with physical examinations, blood draws, laboratory analyses and surveillance imaging. Peritoneal cancer index score (PCI) and completeness of cytoreduction (CCR) were determined intraoperatively by consensus of the operative team.

Patient characteristics (age in years, race, biological sex, American Society of Anesthesiologists (ASA) physical status classification, history of prior laparotomy, history of previous treatment with systemic chemotherapy), disease characteristics (primary site, PCI), pAC tumor grade (low grade: low grade appendiceal mucinous neoplasm (LAMN), high grade: signet ring cell histology), treatment protocol data (time to HIPEC in months, CCR, operative time in minutes, operative blood loss in milliliters (mL), receipt of red blood cell transfusion, receipt of fresh frozen plasma transfusion, multivisceral resection, creation of gastrointestinal anastomoses, creation of ostomy, receipt of adjuvant radiation, receipt of adjuvant chemotherapy) and patient outcomes (intensive care unit (ICU) length of stay (LOS) in days, hospital LOS in days, rates of any complication, rates of 30-day readmission, rates of 30-day perioperative mortality, vital status, disease status at follow-up, disease-specific survival, overall survival, follow-up in months) were abstracted from the medical record.

The patients were stratified by the presence or absence of disease relapse to identify factors associated with the risk of relapse. The patients with disease relapse were then stratified further by receipt of repeat CRS/HIPEC to identify factors associated with receipt of iterative surgical intervention. Primary site-stratified simple logistic regression and multiple logistic regression for overall survival (OS) were performed for the patients with disease relapse after index CRS/HIPEC. All patients received 90-minute HIPEC with mitomycin-C.

### 2.3. Statistical Analysis

GraphPad Prism Version 10.3.1 (464) for macOS was used for all analyses (GraphPad Software, San Diego, CA, USA, www.graphpad.com, accessed on 10 March 2024). Normality of continuous data was determined using the Kolmogorov–Smirnov test. Normally distributed continuous data were reported as the mean with standard deviation (SD) and compared using two-tailed t-tests. Non-normally distributed continuous data were reported as the median with interquartile range (IQR) and compared using Mann–Whitney tests. Categorical data were reported as frequencies and percentages (*n*, %) and compared using chi square tests and Fisher’s exact tests where appropriate. All survival analyses were stratified by disease primary site of origin. Simple logistic regressions were performed for overall survival with odds ratio (OR) and 95% confidence interval (95% CI). Time-to-event analyses for OS and EFS were performed using the Kaplan–Meier method, and the log rank (Mantel–Cox) test was used to determine significant differences between survival curves. *p* values of <0.05 indicated statistical significance.

## 3. Results

### 3.1. Patient and Disease Characteristics

**Figure 1:**
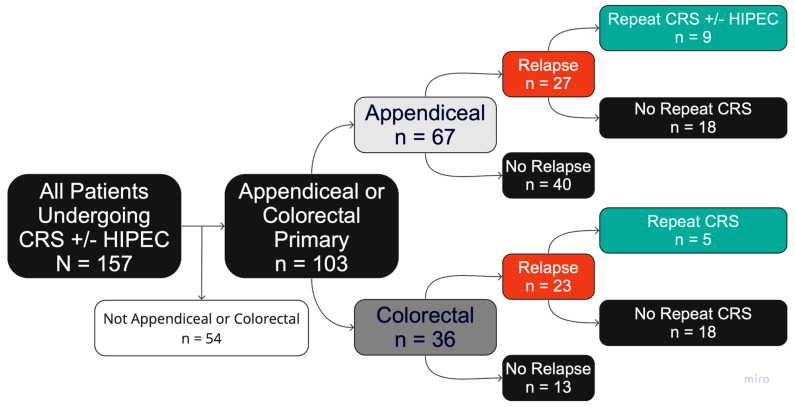
Patient selection flow diagram.

Complete data regarding patient selection and treatment allocations can be found in the flow diagram in Figure 1. A total of 157 patients undergoing CRS/HIPEC were identified. Of these, 54 were excluded for having histologies other than pAC or pCRC. Thus, 103 patients undergoing CRS/HIPEC for pAC or pCRC were identified (*n* = 67 pAC, *n* = 36 pCRC).

Among the 67 patients undergoing CRS/HIPEC for pAC, 27/67 experienced disease relapse (40.3%), and 40/67 had NED at follow-up (59.7%). Of these 27 patients with relapsed pAC, 9/27 underwent repeat CRS/HIPEC (33.3%), and 18/27 did not (66.7%). A total of 9/9 patients undergoing repeat CRS/HIPEC for pAC had low-grade disease (100.0%), and 1/9 had signet ring histology (11.1%). Of the 18 patients with relapsed pAC who did not undergo repeat CRS/HIPEC, 8/18 had low-grade disease (44.4%), 8/18 had high-grade disease (44.4%), and 2/18 had an unknown disease grade (11.1%). Six of these patients (6/18) had signet ring histology (33.3%), and 12/18 had non-signet ring histology (66.6%). Among the 36 patients undergoing CRS/HIPEC for pCRC, 23/36 experienced disease relapse (63.9%), and 13/36 had NED at follow-up (36.1%). Of these 23 patients with relapsed pCRC, 5/23 underwent repeat CRS/HIPEC (21.7%), and 18/23 did not (78.2%).

### 3.2. Disease Relapse

The patients with disease relapse were similar to those without relapse regarding patient age, patient race, patient sex and ASA score (Table 1). When compared to the patients without disease relapse, the patients who relapsed more often had pCRC (46.0% vs. 24.5%, *p* = 0.0224), longer median time to HIPEC in months (6.0 vs. 4.0, *p* = 0.0293) and lower rates of previous receipt of systemic chemotherapy (52.0% vs. 71.7%, *p* = 0.0447). The patients who relapsed also had a higher median PCI (15.5 vs. 8.0, *p* = 0.0266), longer median operative times in minutes (502.3 vs. 407.1, *p* = 0.0004) and higher median operative blood loss in mL (500.0 vs. 250.0, *p* = 0.0006). The rates of RBC transfusion were similar between groups (62.0% vs. 58.5%, *p* = 0.8407), but there were higher rates of FFP transfusion in the relapse group (20.0% vs. 5.7%, *p* = 0.0380). There were no differences between the groups regarding the rates of multivisceral resection, rates of gastrointestinal anastomosis or rates of ostomy creation. The patients who ultimately experienced disease relapse tended to have longer median ICU LOS in days (3.0 vs. 1.0, *p* = 0.0151) but not hospital LOS (8.5 vs. 8.0, *p* = 0.2447). Complication rates were similar between the groups, but the rates of 30-day readmission were higher in the relapse group (36.0% vs. 15.7%, *p* = 0.0238). There were no differences in the rates of 30-day mortality. The rates of adjuvant radiation use were low in both groups (4.0% vs. 1.9%, *p* = 0.6104), but the patients who experienced relapse were significantly more often treated with adjuvant chemotherapy (72.0% vs. 13.2%, *p* < 0.0001). The patients with disease relapse had lower rates of overall survival than those without relapse (46.0% vs. 81.1%, *p* = 0.0002).

### 3.3. Repeat CRS/HIPEC

Among the 50 patients who experienced disease relapse, 14/50 underwent repeat CRS/HIPEC, and 36 did not (Table 2). Overall, those undergoing repeat CRS/HIPEC were similar to those who did not, except for higher rates of female biological sex (92.9% vs. 58.3%, *p* = 0.0211) and a longer median follow-up time in months (51 vs. 16.5, *p* = 0.0003).

### 3.4. Repeat CRS/HIPEC for Relapsed pAC

**Figure 2:**
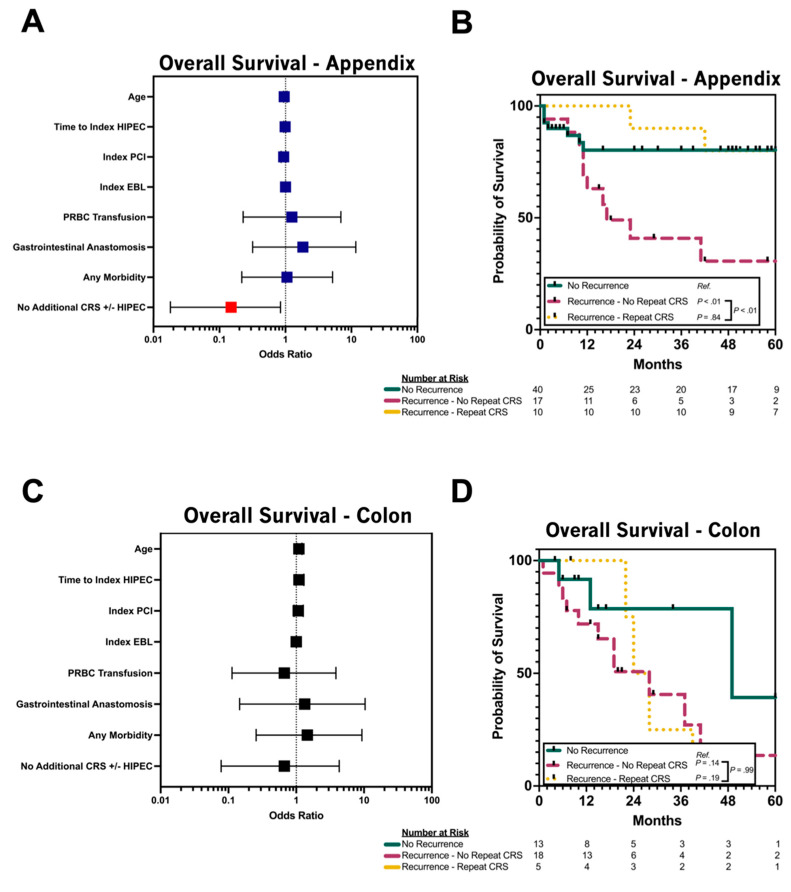
(**A**) Forest plot of overall survival in patients with disease relapse after index HIPEC for metastatic carcinoma of the appendix. (**B**) Overall survival in patients with metastatic carcinoma of the appendix treated with CRS/HIPEC, stratified by disease relapse and implementation of repeat CRS/HIPEC. (**C**) Forest plot of overall survival in patients with disease relapse after index HIPEC for metastatic carcinoma of the colon. (**D**) Overall survival in patients with metastatic carcinoma of the colon treated with CRS + HIPEC, stratified by disease relapse and implementation of repeat CRS/HIPEC. pAC = peritoneal metastases of appendiceal primary; pCRC = peritoneal metastases of colorectal primary; CRS = cytoreductive surgery; HIPEC = hyperthermic intraperitoneal chemotherapy; PCI = peritoneal carcinomatosis index; EBL = estimated blood loss; PRBC = packed red blood cells.

For the patients with relapsed pAC, failing to undergo repeat CRS/HIPEC (OR 0.150; 95% CI 0.018–0.839, *p* = 0.0445) and male patient biological sex (OR 0.119; 0.014–0.689; *p* = 0.0270) were associated with a decreased likelihood of overall survival on univariable analysis (Table 3, Figure 2A). The 3-year OS for the patients with pAC and no relapse was 80.3%. For the patients with recurrent pAC who did not undergo repeat CRS/HIPEC, the 1-year, 3-year and 5-year OSs were 65%, 44.7% and 35.8%, respectively. For the patients with recurrent pAC who underwent repeat CRS/HIPEC, the 1-year, 3-year and 5-year OSs were 88.9%, 88.9% and 77.8%, respectively. For the patients with recurrent pAC, failing to undergo repeat CRS/HIPEC was associated with a significantly decreased OS when compared to the patients who never recurred (log rank *p* = 0.02; Figure 2B). There was no difference in OS between the patients with recurrent pAC who underwent repeat CRS/HIPEC and those who never recurred (log rank *p* = 0.94; Figure 2B). Among the patients with recurrent pAC, those who underwent repeat CRS/HIPEC had a significant improvement in OS (log rank *p* = 0.03; Figure 2B).

### 3.5. Repeat CRS/HIPEC for Relapsed pCRC

For the patients with relapsed pCRC, receipt of additional CRS/HIPEC was not associated with increased odds of OS on simple logistic regression (OR 0.667; 95% CI 0.078–4.291; *p* = 0.6800; Table 4; Figure 2C). The 3-year OS for the patients with pCRC and no relapse was 78.6%. For the patients with recurrent pCRC who did not undergo repeat CRS/HIPEC, the 1-year and 3-year OSs were 71.8% and 40.6%, respectively. For the patients with recurrent pCRC who underwent repeat CRS/HIPEC, the 1-year and 3-year OSs were 100.0% and 25.0%, respectively. For the patients with recurrent pCRC, failing to undergo repeat CRS/HIPEC was associated with a similar OS when compared to the patients who never recurred (log rank *p* = 0.14; Figure 2D). There was no difference in OS between the patients with recurrent pCRC who underwent repeat CRS/HIPEC and those who never recurred (log rank *p* = 0.19; Figure 2D). Among the patients with recurrent pCRC, those who underwent repeat CRS/HIPEC had no significant improvement in OS (log rank *p* = 0.99; Figure 2D).

### 3.6. Subgroup Analysis by Grade and Histology

**Figure 3:**
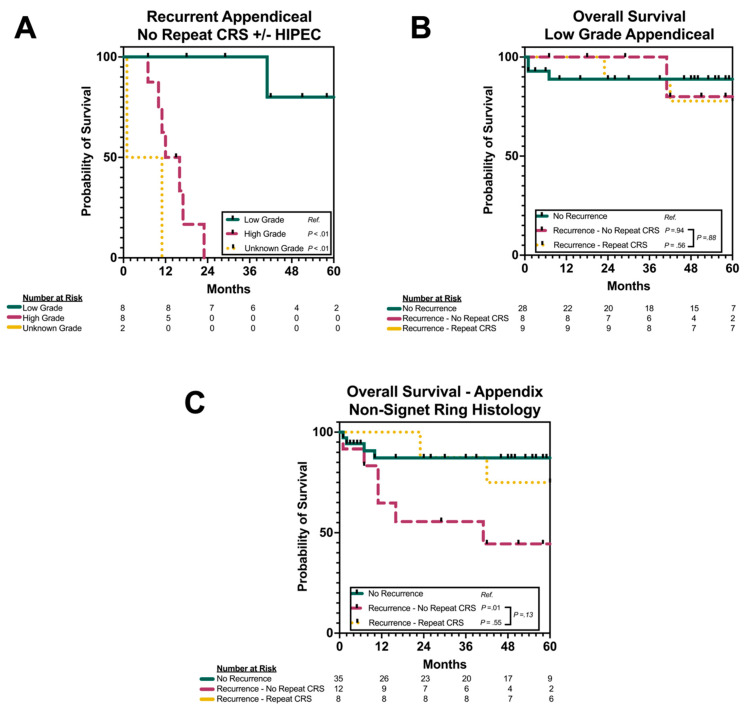
(**A**) Overall survival in patients with recurrent pAC not undergoing repeat CRS/HIPEC, stratified by grade. (**B**) Overall survival in patients with low-grade pAC, stratified by presence or absence of disease recurrence and receipt of repeat CRS/HIPEC. (**C**) Overall survival of patients with non-signet ring histology pAC, stratified by presence or absence of disease recurrence and receipt of repeat CRS/HIPEC. pAC = peritoneal metastases of appendiceal primary; pCRC = peritoneal metastases of colorectal primary; CRS = cytoreductive surgery; HIPEC = hyperthermic intraperitoneal chemotherapy.

Subgroup analysis of the patients with pAC was performed by grade and histology. Among the patients with recurrent pAC not undergoing repeat CRS/HIPEC, the patients with low-grade histology had a 3-year OS of 100.0%. The patients with high-grade histology (*n* = 8) or unknown-grade histology (*n* = 2) had significantly worse OS (both log rank *p* < 0.01; Figure 3A). When examining the patients with low-grade pAC, the patients had similar overall survival whether or not they experienced relapse and whether or not they underwent repeat CRS/HIPEC for their disease relapse (all log rank *p* > 0.05; Figure 3B). When examining all grades of the patients who specifically had non-signet ring histology, the relapsed patients who underwent repeat CRS/HIPEC appeared to have similar survival to those who never recurred (log rank *p* = 0.55), while the patients who recurred and did not undergo repeat CRS/HIPEC had decreased OS (log rank *p* = 0.01, Figure 3C). Given the smaller patient numbers in the pCRC cohort, a similar subgroup analysis was unable to be performed.

## 4. Discussion

Cytoreductive surgery with HIPEC offers prolonged survival for appropriately selected patients with peritoneal surface malignancies, but peritoneal recurrence after CRS/HIPEC is high, greater than 50% in some series, leading others to investigate the role of repeat CRS/HIPEC [19]. As a newer peritoneal surface malignancy program, our series examined the outcomes of a small number of patients with peritoneal recurrence who were managed with repeat CRS/HIPEC and identified comparable outcomes to larger, more established centers.

We identified primary tumor site, receipt of neoadjuvant chemotherapy and burden of disease as risk factors for peritoneal relapse. In terms of safety, operative time, complications and hospital length of stay, there were no significant differences between the initial CRS/HIPEC and repeat CRS/HIPEC. Of the 50 patients who developed relapse after the initial CRS/HIPEC, only a small number (14) underwent repeat CRS/HIPEC. When evaluated based on histology, the pAC patients demonstrated a survival advantage when offered repeat CRS/HIPEC, both for LAMN and high-grade disease, while the pCRC patients did not demonstrate this survival advantage.

The interval to development of peritoneal recurrence and repeat CRS/HIPEC is a potential prognostic indicator of overall outcome. Of the 157 patients initially evaluated in this series, 8.9% of the patients eventually underwent repeat CRS/HIPEC. The median time to repeat CRS/HIPEC was 18 months, and the time to repeat CRS/HIPEC did not negatively impact outcomes for pAC or pCRC pathologies (Table 3 and Table 4). Choudry et al. reported a time interval of 32.2 months between initial and repeat CRS/HIPEC procedures and that multiple CRS/HIPEC procedures were more likely to be performed for peritoneal metastasis from appendiceal cancer and for low and intermediate tumor grade [8]. Konstantinidis and colleagues further elaborated on the impact of time interval and identified that a time interval for repeat CRS/HIPEC of more than 2 years was strongly associated with survival and most pronounced for appendiceal cancer [15].

The results of this series demonstrate similar overall complications after repeat CRS/HIPEC with larger, more established centers. Laks et al. reported a 32% major complication rate after repeat CRS/HIPEC, with 21% requiring reoperation, whereas Konstantinidis and colleagues reported similar morbidity for both initial and repeat CRS/HIPEC procedures (39%, respectively) [9,15]. The overall complication rate was 53% after the initial CRS/HIPEC and 29% after repeat CRS/HIPEC in the current series, and no patients required reoperation. Hospital length of stay was similar between the initial and repeat CRS/HIPEC groups (8 and 7 days, respectively), and readmissions were similar also (14% and 15%, respectively). Major morbidity rates were 39% in the larger US HIPEC collaborative series, and the only perioperative difference in that series was a higher proportion of ostomies placed in the repeat CRS/HIPEC cohort [14].

The optimal outcome of repeat CRS/HIPEC is dependent on tumor biology and optimal cytoreduction [17]. Achieving a complete cytoreduction after prior CRS/HIPEC is challenging, as dense adhesions, recurrent tumor location, distorted anatomy and extensive small bowel mesentery or porta hepatis involvement may contribute to a more difficult operation at repeat CRS/HIPEC. In the Wake Forest experience, the adequacy of cytoreduction was good after repeat CRS/HIPEC, with at least R2a resection status or better achieved in 71% of appendix cancers and 100% of colorectal cancers at the second CRS/HIPEC [12]. The UPMC experience of over 1400 CRS/HIPEC procedures identified similar rates of complete macroscopic resection (CC-0) and optimal cytoreduction (CC-0/1) for the 1st, 2nd and even 3rd repeat CRS/HIPEC procedures (66%, 62% and 70% and 93%, 90% and 94%, respectively), and completeness of cytoreduction was an independent predictor of progression-free survival [8]. The current series achieved complete (CC-0) and optimal cytoreduction (CC-0/1) at the initial CRS/HIPEC in 72% and 94%, respectively, and 78% and 100% of the repeat CRS/HIPEC patients. The US HIPEC Collaborative experience noted that complete cytoreduction rates were less for repeat CRS/HIPEC procedures (83% repeat versus 89% initial CRS/HIPEC) and that repeat CRS/HIPEC procedures were more likely to require complex urologic procedures, such as cystectomy or ureteral resections, and have higher rates of end ileostomy or colostomy placement (10% repeat versus 4.5% initial) [14]. The consistent underlying theme across all centers is that patient selection is perhaps the key metric in identifying those most likely to undergo complete cytoreduction.

There was a clear advantage for patients who developed recurrence after the initial CRS/HIPEC to undergo repeat CRS/HIPEC, and this was most pronounced in pAC pathology. The 1-, 3- and 5-year OSs were significantly improved in relapsed pAC for those who underwent repeat CRS/HIPEC (88.9%, 88.9% and 77.8% versus 65%, 44.7% and 35.8%, respectively). Furthermore, there was no difference in OS for those with recurrent pAC who underwent repeat CRS/HIPEC when compared to those with pAC who never recurred (Figure 2B). These results are congruent with Wake Forest, who identified that appendix cancers had the best prognosis amongst all cancer types after repeat CRS/HIPEC when measuring OS after the initial CRS/HIPEC [12]. Similarly, when evaluating factors associated with survival from the US HIPEC Collaborative, repeat CRS/HIPEC did not affect survival, whereas completeness of cytoreduction and tumor grade were predictive of OS [14].

While admittedly a smaller number of patients, we did not observe the same benefit of repeat CRS/HIPEC for pCRC as others have described. The 1- and 3-year OS for recurrent pCRC patients who did not undergo repeat CRS/HIPEC was 72% and 41%, while the 1- and 3-year OS for those who did undergo repeat CRS/HIPEC was 100% and 25%, respectively. In contradistinction to the pAC patients, the pCRC patients who underwent repeat CRS/HIPEC did not have an improvement of OS (Figure 2B,D). The primary limitation of this subset is the small number of patients and limited follow-up. Others have noted a favorable outcome for patients with recurrent CRC who underwent repeat CRS/HIPEC, with a median survival of 20 months, favoring those with a PCI of 12 or less [20]. Bijelic et al. reported on 26 patients with isolated peritoneal recurrences who underwent repeat CRS/HIPEC, noting a longer median survival than that of those who did not undergo a second operation [21]. More importantly, they cautioned against repeat CRS/HIPEC in patients with diffuse intra-abdominal recurrence, as these patients had a much poorer long-term survival. It is important to note that, while repeat CRS/HIPEC for colorectal cancer is feasible in carefully selected patients, the burden of disease and ability to undergo complete cytoreduction are most notably the important predictors of improved survival [19].

There are limitations to the current study. Importantly, this patient population represents a highly selected, heterogeneous group of patients treated at a single center. The patients were screened and selected for surgery based on a variety of factors, anecdotally with disease-free interval, disease biology and anticipated complete removal of recurrent peritoneal disease being the primary determinant factors. However, it is important to recognize that, when considering surgery, time to recurrence, previous chemotherapy exposure, tumor grade, disease burden, performance status and potential for post-operative morbidity must all be considered in the decision-making process. In addition, there is a significant institutional learning curve and experience that must be achieved in order to reduce perioperative morbidity and improve oncologic outcomes [22]. It is important to note that the decision to offer repeat CRS/HIPEC is often not made in isolation but often based on a multitude of factors. Authors from the United Kingdom select patients who are fit with favorable disease biology, assessing nodal and mutation status, tumor grade as well as disease-free interval as metrics for consideration for repeat CRS/HIPEC [13]. Furthermore, they further stratify based on response to systemic therapy, identifying those with only low volume, intra-abdominal disease who can achieve potential optimal cytoreduction. For patients with marginal performance status, a short disease-free interval, extensive high-grade disease or extra-abdominal recurrence and in whom complete cytoreduction is unachievable, treatment options remain limited. Systemic therapy may provide temporary disease control, but progression of disease is inevitable. It is important to recognize in these instances that patient goals and preferences can help guide decision-making for patients and their caregivers [23].

## 5. Conclusions

In conclusion, repeat CRS/HIPEC for isolated peritoneal recurrence is safe and can offer the potential for long-term survival. Patient selection is key and based on a variety of factors to help guide decision-making and ensure optimal cytoreduction.

## Figures and Tables

**Table 1:** Univariate analysis of patients undergoing HIPEC for metastatic appendiceal or colorectal cancers stratified by disease relapse. pAC = peritoneal metastases of appendiceal primary; pCRC = peritoneal metastases of colorectal primary; CRS = cytoreductive surgery; HIPEC = hyperthermic intraperitoneal chemotherapy; PCI = peritoneal carcinomatosis index; EBL = estimated blood loss; PRBC = packed red blood cells; CCR = completeness of cytoreduction; “SD” = standard deviation; “IQR” = interquartile range.

Variable	Patients Without Relapse (*n* = 53)	Patients with Relapse (*n* = 50)	*p*
Age in Years, mean (SD)	63.2 (13.5)	59.1 (13.2)	0.1229
Race, *n* (%)			0.5225
White	38 (71.7%)	33 (66.0%)	
Black	15 (28.3%)	16 (32.0%)	
Other	0 (0.0%)	1 (2.0%)	
Female Sex, *n* (%)	37 (69.8%)	34 (68.0%)	>0.9999
Primary Site, *n* (%)			0.0224
Appendix	40 (75.5%)	27 (54.0%)	
Colon	13 (24.5%)	23 (46.0%)	
ASA Score			0.1622
2	15 (28.3%)	10 (20.0%)	
3	37 (69.8%)	35 (70.0%)	
4	1 (1.9%)	5 (10.0%)	
Time to HIPEC in Months, median [IQR]	4.0 [3.0–8.0]	6.0 [4–19.0]	0.0293
Prior Laparotomy, *n* (%)	34 (64.2%)	32 (64.0%)	>0.9999
Previous Chemotherapy, *n* (%)	38 (71.7%)	26 (52.0%)	0.0447
Peritoneal Cancer Index Score (PCI), median [IQR]	8.0 [4.0–16.0]	15.5 [6.8–22.3]	0.0266
Cytoreduction Score (CCR), *n* (%)			0.4453
CCR-0	43 (79.6%)	36 (72.0%)	
CCR-1	9 (16.7%)	11 (22.0%)	
CCR-2	1 (1.9%)	3 (6.0%)	
Unknown	1 (1.9%)	0 (0.0%)	
Operative Time in Minutes, mean (SD)	407.1 (109.5)	502.3 (148.3)	0.0004
Operative Blood Loss in mL, median [IQR]	250.0 [125.0–500.0]	500.0 [250.0–850.0]	0.0006
Red Blood Cell Transfusion, *n* (%)	31 (58.5%)	31 (62.0%)	0.8407
Fresh Frozen Plasma Transfusion	3 (5.66%)	10 (20.0%)	0.0380
≥2 Organs Resected, *n* (%)	49 (92.5%)	45 (90.0%)	0.7367
≥4 Organs Resected, *n* (%)	20 (37.7%)	32 (53.3%)	0.1303
≥1 Anastomosis, *n* (%)	43 (81.2%)	38 (76.0%)	0.6323
Ostomy Creation, *n* (%)	13 (24.5%)	19 (38.0%)	0.2009
ICU Length of Stay in Days, median [IQR]	1.0 [1.0–3.0]	3.0 [2.0–4.0]	0.0151
Hospital Length of Stay in Days, [IQR]	8.0 [6.0–10.0]	8.5 [7.0–10.3]	0.2447
Any Complication, *n* (%)	28 (52.8%)	21 (42.0%)	0.3254
Readmission (30 day), *n* (%)	8 (15.7%)	18 (36.0%)	0.0238
Mortality (30 day), *n* (%)	3 (5.7%)	0 (0.0%)	0.2433
Adjuvant Radiation, *n* (%)	1 (1.9%)	2 (4.0%)	0.6104
Adjuvant Chemotherapy, *n* (%)	7 (13.2%)	36 (72.0%)	<0.0001
Follow-Up Status, *n* (%)			<0.0001
No Evidence of Disease	42 (79.2%)	4 (8.0%)	
Alive with Disease	1 (1.9%)	18 (36.0%)	
Died of Disease	2 (3.8%)	27 (54.0%)	
Died of Other Cause/Unknown	8 (15.1%)	1 (2.0%)	
Overall Survival, *n* (%)	43 (81.1%)	23 (46.0%)	0.0002
Follow-Up in Months, median [IQR]	17.0 [6.0–54.0]	21.5 [11.0–42.0]	0.4119

**Table 2:** Univariate analysis of 50 patients with disease relapse stratified by receipt of additional CRS +/− HIPEC. pAC = peritoneal metastases of appendiceal primary; pCRC = peritoneal metastases of colorectal primary; CRS = cytoreductive surgery; HIPEC = hyperthermic intraperitoneal chemotherapy; PCI = peritoneal carcinomatosis index; EBL = estimated blood loss; PRBC = packed red blood cells; CCR = completeness of cytoreduction; “SD” = standard deviation; “IQR” = interquartile range.

Variable	Repeat CRS/HIPEC (*n* = 14)	No Repeat CRS/HIPEC (*n* = 36)	*p*
Age in Years, mean (SD)	63.0 [55.8–71.0]	60.0 [45.0–67.0]	0.5174
Race, *n*%			0.7861
White	9 (64.3%)	24 (66.7%)	
Black	5 (35.7%)	11 (30.6%)	
Other	0 (0.0%)	1 (2.8%)	
Primary Site, *n* (%)			0.3628
Appendix	9 (64.3%)	18 (50.0%)	
Colon	5 (35.7%)	18 (50.0%)	
Female Sex, *n* %	13 (92.9%)	21 (58.3%)	0.0211
ASA Score			0.7117
2	2 (14.3%)	8 (22.2%)	
3	11 (78.6%)	24 (66.7%)	
4	1 (7.1%)	4 (11.1%)	
Time to HIPEC in Months, median [IQR]	18.0 [4.0–26.0]	6.0 [3.3–17.8]	0.4746
Prior Laparotomy, *n* (%)	9 (64.3%)	23 (63.9%)	>0.9999
Previous Chemotherapy, *n* (%)	12 (85.7%)	26 (66.7%)	0.3002
Peritoneal Cancer Index Score (PCI), mean (SD)	16 (10.8)	15.3 (10.4)	0.8285
Cytoreduction Score (CCR), *n* (%)			0.5257
CCR-0	11 (78.6%)	25 (69.4%)	
CCR-1	3 (21.4%)	8 (22.2%)	
CCR-2	0 (0.0%)	3 (8.3%)	
Operative Time in Minutes, mean (SD)	563.1 (157.4)	479 (140.1)	0.0823
Operative Blood Loss in mL, mean (SD)	585.7 (427.6)	673.6 (494.9)	0.5618
Red Blood Cell Transfusion, *n* (%)	8 (57.1%)	23 (63.9%)	0.7500
Fresh Frozen Plasma Transfusion	2 (14.3%)	8 (22.2%)	0.7042
≥2 Organs Resected, *n* (%)	13 (92.9%)	32 (88.9%)	>0.9999
≥4 Organs Resected, *n* (%)	11 (78.6%)	21 (58.3%)	0.1807
≥1 Anastomosis, *n* (%)	9 (64.3%)	29 (80.6%)	0.2777
Ostomy Creation, *n* (%)	2 (14.3%)	17 (47.2%)	0.0504
ICU Length of Stay in Days, median [IQR]	2.0 [1.0–3.5]	3.0 [2.0–4.0]	0.2124
Hospital Length of Stay in Days, [IQR]	7.0 [5.0–9.3]	9.0 [7.3–11.8]	0.0683
Any Complication, *n* (%)	4 (28.6%)	17 (47.2%)	0.3408
Readmission (30 day), *n* (%)	2 (14.3%)	16 (44.4%)	0.0563
Mortality (30 day), *n* (%)	0 (0.0%)	0 (0.0%)	>0.9999
Adjuvant Radiation, *n* (%)	0 (0.0%)	2 (5.6%)	>0.9999
Adjuvant Chemotherapy, *n* (%)	27 (75.0%)	9 (64.3%)	0.49555
Follow-Up Status, *n* (%)			0.1564
No Evidence of Disease	3 (21.4%)	1 (2.8%)	
Alive with Disease	5 (35.7%)	13 (36.1%)	
Died of Disease	6 (42.9%)	21 (58.3%)	
Died of Other Cause/Unknown	0 (0.0%)	1 (2.8%)	
Overall Survival, *n* (%)	8 (57.1%)	14 (38.9%)	0.2430
Follow-Up in Months, median [IQR]	51 [23.8–92.3]	16.5 [10.0–29.0]	0.0003

**Table 3:** Simple logistic regression and multiple logistic regression for overall survival in patients with disease relapse after index HIPEC for metastatic carcinoma of the appendix. pAC = peritoneal metastases of appendiceal primary; pCRC = peritoneal metastases of colorectal primary; CRS = cytoreductive surgery; HIPEC = hyperthermic intraperitoneal chemotherapy; PCI = peritoneal carcinomatosis index; EBL = estimated blood loss; PRBC = packed red blood cells; CCR = completeness of cytoreduction.

Appendix (*n* = 27)						
**Variable**	Simple Logistic Regression			Multiple Logistic Regression		
	OR	95% CI	*p*	OR	95% CI	*p*
Age (per year)	0.995	0.934–1.057	0.0678			
Race						
White	Ref.	Ref.	Ref.			
Black (vs. White)	2.571	0.490–15.91	0.2767			
Sex						
Female	Ref.	Ref.	Ref.	Ref.	Ref.	Ref.
Male (vs. Female)	0.119	0.014–0.689	0.0270	0.137	0.0136–0.906	0.0534
Previous Laparotomy	1.800	0.375–9.123	0.4640			
Time to Index HIPEC (per month)	0.985	0.939–1.016	0.4011			
PCI Score (per point)	0.938	0.858–1.012	0.1216			
Index HIPEC Agent						
Mitomycin C Monotherapy	Ref.	Ref.	Ref.			
Platinum Monotherapy (vs. Mitomycin C)	0.833	0.087–7.992	0.8669			
Duration of Index HIPEC						
90 min	Ref.	Ref.	Ref.			
60 min (vs. 90 min)	0.446	0.084–2.169	0.3237			
Index Operative Time (per minute)	1.001	0.996–1.006	0.7199			
Index EBL (per mL)	1.001	0.999–1.003	0.4975			
Index CCR						
CCR-0	Ref.	Ref.	Ref.			
CCR-1/CCR-2	0.400	0.073–1.973	0.2677			
Intraoperative PRBC Transfusion	1.250	0.228–6.906	0.7933			
Intraoperative FFP Transfusion	0.800	0.145–4.380	0.7933			
Multivisceral Resection	1.200	0.125–11.54	0.8669			
Gastrointestinal Anastomosis	1.833	0.318–11.64	0.4978			
Intensive Care Unit Length of Stay (per day)	0.934	0.676–1.052	0.4336			
Hospital Length of Stay (per day)	0.947	0.806–1.034	0.3497			
Any Morbidity	1.050	0.217–5.155	0.9512			
No Additional CRS/HIPEC	0.150	0.018–0.839	0.0445	0.175	0.017–1.167	0.0906

**Table 4:** Simple logistic regression for overall survival in patients with disease relapse after index HIPEC for metastatic carcinoma of the colon. pAC = peritoneal metastases of appendiceal primary; pCRC = peritoneal metastases of colorectal primary; CRS = cytoreductive surgery; HIPEC = hyperthermic intraperitoneal chemotherapy; PCI = peritoneal carcinomatosis index; EBL = estimated blood loss; PRBC = packed red blood cells.

**Colon (*n* = 23)**			
**Variable**	**Simple Logistic Regression**		
	**OR**	**95% CI**	***p***
Age (per year)	1.094	1.014–1.213	0.0420
Time to Index HIPEC (per month)	1.099	0.996–1.294	0.0685
PCI Score (per point)	1.071	0.927–1.265	0.3736
Index Operative Time (per minute)	1.002	0.993–1.011	0.6583
Index EBL (per mL)	1.000	0.999–1.003	0.6110
Intraoperative PRBC Transfusion	0.667	0.113–3.841	0.6457
Gastrointestinal Anastomosis	1.333	0.146–10.36	0.7822
Intensive Care Unit Length of Stay (per day)	1.208	0.768–2.482	0.5094
Hospital Length of Stay (per day)	1.107	0.883–1.515	0.4366
Any Morbidity	1.458	0.256–9.312	0.6734
No Additional CRS/HIPEC	0.667	0.078–4.291	0.6800

## Data Availability

Data presented in this study are available in this article.

## References

[1] Levine E.A., Stewart J.H., Shen P., Russell G.B., Loggie B.L., Votanopoulos K.I. (2014). Intraperitoneal chemotherapy for peritoneal surface malignancy: Experience with 1000 patients. J. Am. Coll. Surg..

[2] Chua T.C., Moran B.J., Sugarbaker P.H., Levine E.A., Glehen O., Gilly F.N., Baratti D., Deraco M., Elias D., Sardi A. (2012). Early- and long-term outcome data of patients with pseudomyxoma peritonei from appendiceal origin treated by a strategy of cytoreductive surgery and hyperthermic intraperitoneal chemotherapy. J. Clin. Oncol..

[3] Verwaal V.J., Bruin S., Boot H., van Slooten G., van Tinteren H. (2008). 8-year follow-up of randomized trial: Cytoreduction and hyperthermic intraperitoneal chemotherapy versus systemic chemotherapy in patients with peritoneal carcinomatosis of colorectal cancer. Ann. Surg. Oncol..

[4] Verwaal V.J., Boot H., Aleman B.M.P., van Tinteren H., Zoetmulder F.A.N. (2004). Recurrences after peritoneal carcinomatosis of colorectal origin treated by cytoreduction and hyperthermic intraperitoneal chemotherapy: Location, treatment, and outcome. Ann. Surg. Oncol..

[5] Bijelic L., Yan T.D., Sugarbaker P.H. (2008). Treatment failure following complete cytoreductive surgery and perioperative intraperitoneal chemotherapy for peritoneal dissemination from colorectal or appendiceal mucinous neoplasms. J. Surg. Oncol..

[6] Chua T.C., Liauw W., Morris D.L. (2012). Early recurrence of pseudomyxoma peritonei following treatment failure of cytoreductive surgery and perioperative intraperitoneal chemotherapy is indicative of a poor survival outcome. Int. J. Colorectal. Dis..

[7] Chu D.Z., Lang N.P., Thompson C., Osteen P.K., Westbrook K.C. (1989). Peritoneal carcinomatosis in nongynecologic malignancy. A prospective study of prognostic factors. Cancer.

[8] Choudry H.A., Bednar F., Shuai Y., Jones H.L., Pai R.K., Pingpank J.F., Ahrendt S.S., Holtzman M.P., Zeh H.J., Bartlett D.L. (2019). Repeat Cytoreductive Surgery-Hyperthermic Intraperitoneal Chemoperfusion is Feasible and Offers Survival Benefit in Select Patients with Peritoneal Metastases. Ann. Surg. Oncol..

[9] Laks S., Schtrechman G., Adileh M., Ben-Yaacov A., Purim O., Ivanov V., Aderka D., Shacham-Shmueli E., Halpern N., Goren S. (2021). Repeat Cytoreductive Surgery and Intraperitoneal Chemotherapy for Colorectal Cancer Peritoneal Recurrences is Safe and Efficacious. Ann. Surg. Oncol..

[10] Williams B.H., Alzahrani N.A., Chan D.L., Chua T., Morris D. (2014). Repeat cytoreductive surgery (CRS) for recurrent colorectal peritoneal metastases: Yes or no?. Eur. J. Surg. Oncol..

[11] Karpes J.B., Lansom J.D., Alshahrani M., Parikh R., Shamavonian R., A Alzahrani N., Liauw W., Morris D.L. (2020). Repeat cytoreductive surgery with or without intraperitoneal chemotherapy for recurrent epithelial appendiceal neoplasms. BJS Open.

[12] Valenzuela C.D., Levine E.A., Mangieri C.W., Gawdi R., Moaven O., Russell G., Lundy M.E., Perry K.C., Votanopoulos K.I., Shen P. (2022). Repeat Cytoreductive Surgery with Hyperthermic Intraperitoneal Chemotherapy for Cancers with Peritoneal Metastasis: A 30-year Institutional Experience. Ann. Surg. Oncol..

[13] Sutton P.A., O’Dwyer S.T., Barriuso J., Aziz O., Selvasekar C., Renehan A., Wilson M. (2021). Indications and outcomes for repeat cytoreductive surgery and heated intra-peritoneal chemotherapy in peritoneal surface malignancy. Surg. Oncol..

[14] Powers B.D., Felder S., Veerapong J., Baumgartner J.M., Clarke C., Mogal H., Staley C.A., Maithel S.K., Patel S., Dhar V. (2020). Repeat Cytoreductive Surgery and Hyperthermic Intraperitoneal Chemotherapy Is Not Associated with Prohibitive Complications: Results of a Multiinstitutional Retrospective Study. Ann. Surg. Oncol..

[15] Konstantinidis I.T., Levine E.A., Chouliaras K., Russell G., Shen P., Votanopoulos K.I. (2017). Interval between cytoreductions as a marker of tumor biology in selecting patients for repeat cytoreductive surgery with hyperthermic intraperitoneal chemotherapy. J. Surg. Oncol..

[16] Feferman Y., Solomon D., Bhagwandin S., Kim J., Aycart S.N., Feingold D., Sarpel U., Labow D.M. (2019). Sites of Recurrence After Complete Cytoreduction and Hyperthermic Intraperitoneal Chemotherapy for Patients with Peritoneal Carcinomatosis from Colorectal and Appendiceal Adenocarcinoma: A Tertiary Center Experience. Ann. Surg. Oncol..

[17] Votanopoulos K.I. (2022). Repeat CRS/HIPEC: It Comes Down to Tumor Biology and Ability to Achieve a Complete CRS. Ann. Surg. Oncol..

[18] Vandenbroucke J.P., von Elm E., Altman D.G., Gøtzsche P.C., Mulrow C.D., Pocock S.J., Poole C., Schlesselman J.J., Egger M. (2007). Strengthening the Reporting of Observational Studies in Epidemiology (STROBE): Explanation and elaboration. Ann. Intern. Med..

[19] Mogal H., Chouliaras K., Levine E.A., Shen P., Votanopoulos K.I. (2016). Repeat cytoreductive surgery with hyperthermic intraperitoneal chemotherapy: Review of indications and outcomes. J. Gastrointest. Oncol..

[20] Portilla A.G., Sugarbaker P.H., Chang D. (1999). Second-look surgery after cytoreduction and intraperitoneal chemotherapy for peritoneal carcinomatosis from colorectal cancer: Analysis of prognostic features. World J. Surg..

[21] Bijelic L., Yan T.D., Sugarbaker P.H. (2007). Failure analysis of recurrent disease following complete cytoreduction and perioperative intraperitoneal chemotherapy in patients with peritoneal carcinomatosis from colorectal cancer. Ann. Surg. Oncol..

[22] Polanco P.M., Ding Y., Knox J.M., Ramalingam L., Jones H., Hogg M.E., Zureikat A.H., Holtzman M.P., Pingpank J., Ahrendt S. (2015). Institutional learning curve of cytoreductive surgery and hyperthermic intraperitoneal chemoperfusion for peritoneal malignancies. Ann. Surg. Oncol..

[23] Wall J.A., Pozzar R.A., Enzinger A.C., Tavormina A., Howard C., Matulonis U.A., Liu J.F., Horowitz N., Meyer L.A., Wright A.A. (2024). Improving the palliative-procedure decision-making process for patients with peritoneal carcinomatosis: A secondary analysis. Gynecol. Oncol..

